# Supplementary material for: Multiphoton Microscopy for Identifying Collagen Signatures Associated with Biochemical Recurrence in Prostate Cancer Patients
Source: J Pers Med. 2021 Oct 22;11(11):1061. doi: 10.3390/jpm11111061 (PMC8619628; doi:10.3390/jpm11111061)
Supplement: Supplementary file 1 [file jpm-11-01061-s001.zip › jpm-1366670-supplementary.pdf]

Supplementary Materials

Table S1. Baseline clinical characteristics of patients treated with radical prostatectomy and with MPM-identified collagen features from prostatectomy specimen.

**Variable**	**Overall (n= 59)**
Age at surgery (yr)	62 (58.5,67.0)
Race, n (%)	
Other	48 (0.81)
African American	11 (0.19)
PSA (ng/ml)	5.4 (4.2, 8.5)
Final Pathology Stage, n (%)	
pT2	38 (0.64)
pT3a	13 (0.22)
pT3b	8 (0.14)
Final Pathology Grade Group, n (%)	
GG 1	5 (0.08)
GG 2	30 (0.51)
GG 3	18 (0.31)
GG 4/5	6 (0.10)
Extraprostatic extension, n (%)	
Absent	42 (0.71)
Present	17 (0.29)
Seminal vesicular invasion, n (%)	
Absent	53 (0.90)
Present	6 (0.10)
Lymphovacular invasion, n (%)	
Absent	55 (0.93)
Present	4 (0.07)
Positive surgical margin, n (%)	
Absent	54 (0.92)
Present	5 (0.08)

Table S2. Label-free MPM profiling and quantification of prostate stromal composition

MPM Image Channel	Contrast Source	Quantifiers	Software
SHG Red channel	Fibrillar collagen	**AF**<br>Fraction of tissue occupied by SHG-emitting collagen<br>Measures the amount of collagen in an ROI	Fiji
		**I_R**<br>Mean SHG signal intensity<br>Measures the brightness of collagen fibers in a ROI	Fiji
		**Fiber width, length, angle**<br>Mean width and length of SHG-emitting fibers<br>Measures individual fiber morphology in a ROI	CT-FIRE Curve Align
		**Fiber orientation**<br>Bulk coherence of SHG-emitting fibers<br>Measures the degree of collagen alignment in a ROI	Curve Align
2PAF Green channel	Smooth muscle Stromal cells	**I_G**<br>Mean 2PAF signal intensity<br>Measures the brightness of tissue autofluorescence	Fiji
SHG/2PAF Red and green channels	Collagen and stromal cells	**$I_R/(I_R+I_G)$**<br>Normalized SHG signal intensity<br>Measures brightness of collagen fibers relative to tissue autofluorescence	Fiji

Table S3. Summary of image analysis steps for each region of interest.

**Step 1: Image Possessing**		**Software**
1a	Background subtraction	Fiji
1b	MPM image registration on reference H&E	MATLAB
1c	Tumor area annotation and region of interest selection	QuPath
**Step 2: Image Segmentation**		
2a	Stromal to tumor glands boundary creation	CurveAlign
2b	Stromal region segmentation	CurveAlign
**Step 3: Collagen Area Fraction and Intensity Quantification**		
3a	Thresholding of SHG and 2PAF channel	Fiji
3b	Pixel area and mean pixels intensity calculations per channel	Fiji
**Step 4: Collagen Fiber Features Quantification**		
4a	Segmentation and extraction of individual fibers	CT-FIRE
4b	Individual fiber width and length calculation	CT-FIRE
4c	Mean value calculation for each quantifier	CT-FIRE
**Step 5: Collagen Orientation Calculations**		
5a	Stromal to tumor glands boundary creation	CurveAlign
5b	Fiber Angle orientation relative to the boundary calculation	CurveAlign
5c	Mean value calculations	CurveAlign

Table S4. Univariable Cox Proportional Hazards models to evaluate association of time to biochemical recurrence and clinicopathological variables in the prostatectomy cohort. All variables, except age and PSA were factored. Age and PSA were evaluated as continuous variables. HR- Hazard Ratio, CI-Confidence Interval.

Variable	Univariable analysis			
	HR	95% CI		p value >|z|
Age (per year)	1.01	0.93	1.10	0.797
PSA (ng/ml)	1.02	0.97	1.08	0.405
Final Pathology Grade Group				
1-2	ref			
3	15.66	1.98	123.77	0.009
4-5	5.94	0.37	94.98	0.208
Final Pathology Stage				
pT2	ref			
pT3a	1.23	0.23	6.73	0.811
pT3b	7.85	2.09	29.39	0.002
Extraprostatic Extension				
Absent	ref			
Present	2.66	0.81	8.72	0.107
Seminal Vesicular Invasion	ref			
Absent				
Present	6.07	1.77	20.79	0.004
Positive Surgical Margin				
Absent	ref			
Present	2.22	0.48	10.30	0.308

Table S5. A multivariable Cox Proportional Hazards model to evaluate association of time to biochemical recurrence and selected collagen variables in the prostatectomy cohort. Only variables with $p < 0.05$ on univariable analysis were included. Interdependent collagen variables, such as collagen area fraction, intensities and fiber width were not included in the same model. HR- Hazard Ratio, CI-Confidence Interval, RP-Radical Prostatectomy.

Variable	Multivariable analysis			
	HR	95% CI		p value >|z|
Collagen Fiber Intensity (I_R)	1.06	1.02	1.09	0.004
Collagen Fiber Angle	2.75	1.34	5.64	0.006
RP Grade Group				
1-2	ref			
3	1.17	1.03	99.67	0.047
4-5	2.64	0.00	16.62	0.370
RP Stage	0.89	0.08	2.52	0.357
Seminal Vesicular Invasion	2.24	1.57	10284.54	0.031

Table S6. Univariable Cox Proportional Hazards models to evaluate association of time to biochemical recurrence and collagen variables in patients at intermediate risk (RP Grade Group of 2 and 3) from the prostatectomy cohort.

Variable	Univariable analysis			
	HR	95% CI		p value >|z|
Collagen Area Fraction (AF)	1.05	1.01	1.09	0.019
Collagen Fiber Intensity (I_R)	1.02	1.01	1.04	0.009
Normalized Intensity (I_R/I_R+I_G)	1.13	1.04	1.22	0.004
Collagen Fiber Lenght (μm)	1.10	0.97	1.25	0.154
Collagen Fiber Width (μm)	3.02	1.03	8.87	0.044
Collagen Fiber Angle (degrees)	1.25	0.87	1.77	0.224
Collagen Fiber Coherence	0.95	0.89	1.01	0.122

Table S7. Univariable Cox Proportional Hazards models to evaluate association of time to biochemical recurrence and selected clinical variables in the biopsy cohort. All variables, except age and PSA were factored.

Variable	Univariable analysis			
	HR	95% CI		p value >|z|
Age (per year)	1.02	0.93	1.12	0.678
PSA (ng/ml)	1.12	1.04	1.20	0.002
Biopsy Grade Group				
1-2	ref			
3	12.10	1.31	112.05	0.028
4-5	11.11	1.21	102.44	0.034
Clinical Stage				
cT1	ref			
cT2	1.59	0.42	5.94	0.493

Table S8. Multivariable Cox Proportional Hazards model to evaluate association of time to biochemical recurrence and collagen variables in the biopsy cohort. Only variables with $p < 0.05$ on univariable analysis were included. Interdependent collagen variables, such as collagen area fraction, intensities and fiber width were not included in the same model.

Variable	Multivariable analysis			
	HR	95% CI		p value >|z|
Collagen Fiber Intensity (I_R)	1.10	1.01	1.19	0.020
PSA (ng/ml)	1.11	1.01	1.22	0.036
Biopsy Grade Group				
1-2	ref			
3	7.62	0.46	126.88	0.157
4-5	4.70	0.28	78.40	0.281
